# Delayed graft function and long-term outcomes after deceased donor kidney transplantation: insights from mate-kidney analysis

**DOI:** 10.3389/ti.2026.16526

**Published:** 2026-06-16

**Authors:** Oscar A. Garcia Valencia, Ahmed Zeen Alabedeen Alrifai, Mohammed Y. Mahgoub, Girish Mour, Byron Smith, Andrew J. Bentall, Naim S. Issa, Caroline C. Jadlowiec, Alexander R. Cortez, Hatem Amer, Samy Riad

**Affiliations:** 1 Department of Medicine, Division of Nephrology and Hypertension, Mayo Clinic, Rochester, MN, United States; 2 Department of Medicine, Division of Nephrology and Hypertension, Mayo Clinic, Scottsdale, AZ, United States; 3 Department of Biomedical Statistics and Informatics, Mayo Clinic, Rochester, MN, United States; 4 Department of Surgery, Division of Transplantation Surgery, Mayo Clinic, Scottsdale, AZ, United States; 5 Department of Surgery, Division of Transplantation Surgery, Mayo Clinic, Rochester, MN, United States

**Keywords:** allocation policy change, delayed graft function, long-term outcomes, mate kidney, rejection

## Abstract

Delayed graft function (DGF) is a frequent complication after deceased donor kidney transplantation, yet its long-term impact remains understudied. Heterogeneous populations, short follow-up, and inadequate control of donor-related factors have limited prior studies. Using the Scientific Registry of Transplant Recipients (2005–2024), we evaluated the association between DGF and long-term graft outcomes, including a mate-kidney (same donor, discordant DGF status) analysis to isolate its independent effect. We identified 103,678 dialysis-dependent deceased donor kidney recipients maintained on tacrolimus and mycophenolate. Mixed-effects Cox models, adjusted for donor, recipient, immunologic, and procurement factors. DGF occurred in 30.1% of recipients and was associated with increased death-censored graft failure (HR 1.40; 95% CI 1.33–1.47) and overall graft failure (HR 1.27; 95% CI 1.23–1.31). DGF recipients had higher rejection rates at 6 (6.0% vs. 4.7%) and 12 months (8.9% vs. 7.3%), longer hospital stay, and higher 1-year rehospitalization. In the mate-kidney analysis (6,818 donors), DGF remained strongly associated with death-censored graft failure (HR 1.58; 95% CI 1.39–1.80) and overall graft failure (HR 1.35; 95% CI 1.25–1.46), confirming the independent effect of DGF. DGF is a strong predictor of adverse long-term outcomes irrespective of rejection and warrants targeted prevention strategies and intensified follow-up.

## Graphical Abstract

**Figure:**
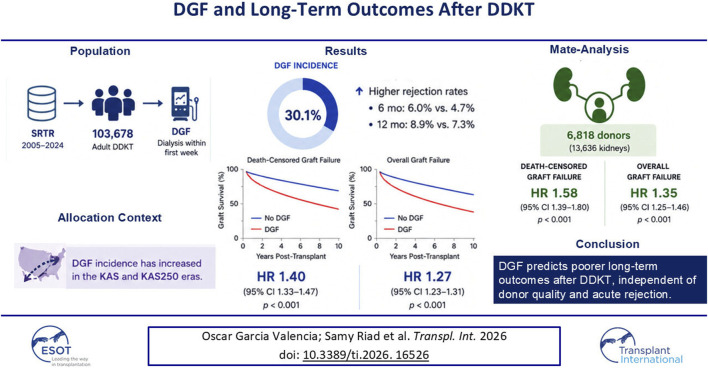

## Highlights

- Delayed graft function is associated with significantly increased long-term risks of both death-censored and overall kidney graft failure.
- This association persists independent of acute rejection and after controlling for donor characteristics using mate-kidney analysis, confirming that delayed graft function itself, rather than donor quality, is a key determinant of long-term outcomes.
- The rising incidence of DGF in the KAS and KAS250 eras underscores important clinical and policy implications in the current allocation landscape.

## Introduction

Delayed graft function (DGF), commonly defined as the need for dialysis within the first week after kidney transplantation, is a frequent complication after deceased donor kidney transplantation [1], affecting approximately 25%–35% of recipients [2]. Despite substantial advances in organ preservation, perioperative care, and immunosuppression, DGF remains a major clinical and operational challenge.

Short- and intermediate-term consequences of DGF, including longer hospital stays, higher healthcare costs, increased rejection, and reduced 1–5-year graft survival are well documented. However, the long-term impact of DGF beyond 5 years remains poorly understood, with prior studies limited by small sample sizes, heterogeneous immunosuppression, and inadequate control for donor factors that strongly influence graft survival [3–6].

Delayed graft function arises from a complex interplay of donor-, recipient-, and procurement-level factors, including ischemia-reperfusion injury, donor quality, cold ischemia time, and recipient comorbidity. Because donor characteristics substantially affect both early graft function and long-term outcomes, distinguishing the independent effect of DGF from donor quality is challenging [7, 8]. Mate-kidney analysis, which compares outcomes between kidneys from the same donor with discordant for DGF status, is a powerful approach to overcoming this confounding but has rarely been applied in studies with long follow-up.

In parallel, kidney allocation in the United States has undergone substantial changes over the past decade. Prior to 2014, allocation was largely based on donation service areas and waiting time, contributing to geographic disparities. The Kidney Allocation System (KAS) [9], implemented in 2014 and revised in 2021 (KAS250) [10], introduced prioritization based on recipient and donor characteristics and expanded geographic sharing through a 250–nautical–mile radius. These changes have influenced donor utilization and transplant logistics, with potential downstream effects on delayed graft function and long-term transplant outcomes.

To address these gaps, we conducted a national analysis of all primary dialysis-dependent deceased donor kidney transplants in the United States between 2005 and 2024. Our objectives were to:

1. Evaluate the association between DGF and long-term death-censored and overall graft failure, and
2. Perform a mate-kidney analysis to isolate the independent effect of DGF from donor-related factors.

## Materials and methods

### Data source

This study used data from the Scientific Registry of Transplant Recipients (SRTR). The SRTR data system includes data on all donors, wait-listed candidates, and transplant recipients in the US, submitted by the members of the Organ Procurement and Transplantation Network (OPTN). The Health Resources and Services Administration (HRSA), U.S. Department of Health and Human Services provide oversight to the activities of the OPTN and SRTR contractors.

### Study population

Using the Scientific Registry of Transplant Recipients standard analysis file, we identified all dialysis-dependent primary deceased donor kidney recipients (N = 173,860) between 2005 and 2024. We excluded recipients discharged on maintenance immunosuppression other than tacrolimus and mycophenolate with or without steroids (n = 22,026). Moreover, we excluded recipients of no induction or mixed induction (n = 28,965) and those with a positive or unknown crossmatch (n = 19,191). Thus, our cohort consisted of 103,678 recipients and was divided into two groups of recipients based on the DGF status: the DGF group (n = 31,197) and the No-DGF group (n = 72,481). Delayed graft function (DGF) was defined according to the SRTR/OPTN registry criteria as the need for at least one dialysis treatment within the first week after kidney transplantation.

**FIGURE 1:**
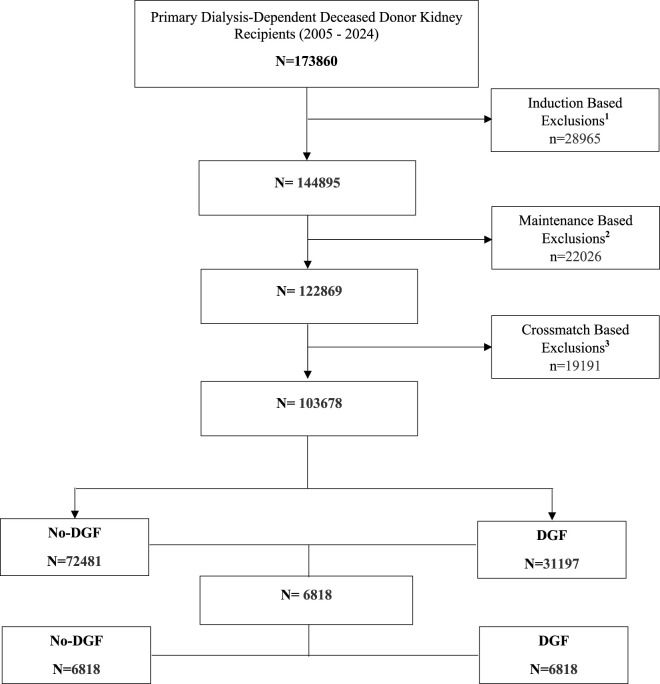
Study Population. 1. Induction-based exclusions: use of unusual agents, combination agents, missing or no-induction. 2. Maintenance-based exclusions: missing immunosuppression or regimens other than Tacrolimus/MMF with or without steroids. 3. Missing, positive, or weakly positive crossmatch.

An additional mate-kidney analysis was performed among recipients with kidneys from the same donor (i.e., mate kidneys) (n = 13,636 kidneys from 6,818 donors). Pairs of kidneys from the same donor who met the above inclusion criteria and were identified where one recipient experienced DGF while the other did not (Figure 1).

### Outcomes of interest

The primary outcome of the study was death-censored kidney graft failure and overall graft failure by recipient’s DGF status, with overall graft failure defined as graft loss or death. Secondary outcomes included 6-month and 1-year rejection rate, length of stay, rehospitalization within the first year, BK polyoma virus infection rate, and 1 year creatinine according to recipient DGF status.

### Statistical analyses

Continuous variables were summarized with the mean and standard deviation. Differences between groups were tested using analysis of variance or pooled t-tests. For categorical variables, data were summarized as counts and within-group percentages. Proportions were compared by the chi-square test. Time to event data were summarized with Kaplan-Meier estimates of incidence and curves for death-censored and overall graft survival through 10 years post-transplant. We left-censored the cohort at 90 days to limit the contribution of early complications, including primary non-function and death. Log-rank tests were used to identify statistically significant differences between groups.

Mixed effect Cox proportional hazard models examined the association between DGF and outcomes of interest. Models were adjusted for variables selected *a priori* based on medical relevance after review by the study team including donor (age, ethnicity, sex, DBD vs. DCD, KDRI), recipient (age, ethnicity, sex, ESKD etiology, dialysis vintage, presence of vascular disease, induction type and maintenance), immunologic (HLA mismatch, cPRA) and procurement factors (preservation). Transplant center was used as a random effect in these models. For the mate analysis, a random effect was also used for the donor. Linearity in all tests was evaluated using splines for continuous variables. Plots of Schoenfeld residuals were used to test the assumption of proportionality. We also performed sensitivity analyses on the full cohort, without left censoring, to assess the robustness of our findings.

To further evaluate the relationship between delayed graft function (DGF) and acute rejection, we performed additional analyses stratified by rejection status within the first-year post-transplant. Separate Cox proportional hazards models were constructed among recipients with and without rejection. In addition, an interaction term between DGF and rejection was included in the full cohort model to assess for effect modification. All models were adjusted using the same covariates as the primary analysis. All analyses were performed using R v4.2.2 (R Foundation for Statistical Computing, Vienna, Austria). P-values less than 0.05 were considered statistically significant.

## Results

### Baseline characteristics

Our cohort consisted of 103, 678 deceased donor kidney transplant recipients of whom 72,481 (69.90%) recipients did not experience DGF and 31,197 (30.09%) did. Recipients with DGF were older (54.8 vs. 52.7 years, p < 0.001), less likely to be women (31.8% vs. 41.5%, p < 0.001) and more likely to be Black (41.5% vs. 35.4%, p < 0.001). Diabetes mellitus as an etiology of end-stage kidney disease was more frequently observed in recipients with DGF (40.1% vs. 29.8%, p < 0.001), while glomerulonephritis (15.4% vs. 20.4%, p < 0.001) was less frequently observed compared to recipients without DGF. Recipients with DGF had longer waiting time on dialysis and had a higher KDRI (Table 1).

**TABLE 1:** Baseline characteristics of recipients by delayed graft function status.

Variable	No DGF N = 72,481	DGF N = 31,197	p value
Recipient variables			
Recipient age	52.7 (13.3)	54.8 (12.2)	<0.001
Recipient sex (female)	30,092 (41.5%)	9,922 (31.8%)	<0.001
Ethnicity	​	​	<0.001
Black	25,648 (35.4%)	12,950 (41.5%)	​
Hispanic	13,183 (18.2%)	6,076 (19.5%)	​
Other	6,993 (9.6%)	3,142 (10.1%)	​
White	26,657 (36.8%)	9,029 (28.9%)	​
ESKD etiology	​	​	<0.001
DM	21,593 (29.8%)	12,515 (40.1%)	​
GN	14,757 (20.4%)	4,809 (15.4%)	​
Other	30,236 (41.7%)	11,981 (38.4%)	​
PKD	5,881 (8.1%)	1882 (6.0%)	​
Recipient BMI	28.3 (5.41)	29.6 (5.49)	<0.001
Time on dialysis (years)	4.26 (3.1)	5.04 (3.2)	<0.001
Recipient vascular disease	7,743 (18.7%)	3,862 (23.2%)	<0.001
Donor variables			
Donor age	38.1 (16.1)	42.0 (14.7)	<0.001
Donor Race	​	​	<0.001
Black	10,675 (14.7%)	4,306 (13.8%)	​
Hispanic	10,273 (14.2%)	4,114 (13.2%)	​
Other	2,564 (3.5%)	1,248 (4.0%)	​
White	48,969 (67.6%)	21,529 (69.0%)	​
Donor sex (female)	28,995 (40.0%)	11,248 (36.1%)	<0.001
Donor cause of death	​	​	<0.001
Anoxia	25,652 (35.4%)	12,811 (41.1%)	​
Cerebrovascular	19,957 (27.5%)	9,316 (29.9%)	​
Head trauma	24,434 (33.7%)	7,892 (25.3%)	​
Other	2,438 (3.4%)	1,178 (3.8%)	​
Donor diabetes	24,274 (34.3%)	13,761 (45.4%)	<0.001
KDRI	1.130 (0.284)	1.198 (0.274)	<0.001
Procurement variables			
DCD	12,147 (16.8%)	10,124 (32.5%)	<0.001
Pumped	33,453 (46.2%)	16,087 (51.6%)	<0.001
Cold ischemia time (hrs.)	17.4 (8.0)	19.9 (8.4)	<0.001
Laterality	​	​	<0.001
Left	33,349 (46.0%)	14,516 (46.5%)	​
Right	36,929 (50.9%)	15,945 (51.1%)	​
*En-bloc*	1,500 (2.1%)	357 (1.1%)	​
Sequential	703 (1.0%)	379 (1.2%)	​
Inotropic support (yes)	29,974 (41.4%)	10,694 (34.3%)	<0.001
Local organ	51,734 (71.4%)	19,565 (62.7%)	<0.001
Immunologic variables			
cPRA	20.3% (32.8)	18.2% (30.6)	<0.001
HLA-mismatch	​	​	<0.001
0	4,442 (6.1%)	1,283 (4.1%)	​
1–3	13,395 (18.5%)	5,208 (16.7%)	​
4–6	54,642 (75.4%)	24,706 (79.2%)	​
Induction	​	​	<0.001
Alemtuzumab	11,171 (15.4%)	4,933 (15.8%)	​
IL-2RA	14,344 (19.8%)	5,576 (17.9%)	​
rATG	46,966 (64.8%)	20,688 (66.3%)	​
Steroid maintenance	49,825 (68.7%)	23,138 (74.2%)	<0.001

Values are shown as mean (SD) for continuous variables and n (%) for categorical variables. P-values were calculated using t-tests or ANOVA, for continuous variables and chi-square tests for categorical variables.

Abbreviations: BMI, body mass index; ESKD, end-stage kidney disease; PCKD, polycystic kidney disease; PVD, peripheral vascular disease; HLA, human leukocyte antigen; cPRA, calculated panel reactive antibody; IL-2RA, interleukin-2 receptor antagonist.

Recipients who experienced DGF were more likely to have received kidneys from older donors (42.0 vs. 38.1 years p < 0.001), donation after cardiac death (DCD) donors (DCD, 32.5% vs.16.8%, p < 0.001), and imported kidneys (37.3% vs. 28.6%, p < 0.001). These kidneys had longer cold ischemia times (19.9 vs. 17.4 h, p < 0.001), and their donors were less frequently supported with inotropic agents before donation (34.3% vs. 41.4%, p < 0.001). A higher number of HLA-mismatches was noted among the recipients with DGF. The induction type was similar between the two groups (Table 1).

### Unadjusted outcomes

**FIGURE 2:**
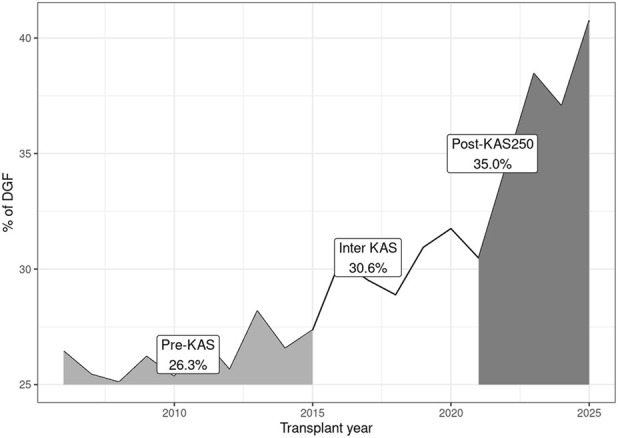
Trends in delayed graft function (DGF) across allocation eras. DGF rates increased from approximately 26% in the pre-KAS era to over 35% following the implementation of KAS250. The y-axis is not set to zero to better visualize temporal trends within the observed range.

The DGF rate increased over the study period. Before the KAS was implemented in 2014, the DGF rate averaged around 26.3%, while after KAS the DGF rate averaged around 30.6%. In March of 2021, KAS250 was implemented, and the rate of DGF has since averaged 35.0% (Figure 2). The increase in DGF rates over time was most pronounced following implementation of the KAS and KAS250 allocation policies, with a relative increase of approximately 16% from the pre-KAS to post-KAS250 era. This trend likely reflects evolving donor utilization practices, including increased use of donation after circulatory death (DCD) donors and longer ischemia times.

On univariable analysis, DGF was associated with worse short-term outcomes (Table 2). The length of stay was longer in the DGF group (8.2 days, vs. 5.7 days) compared to the recipients without DGF (p < 0.001). The rejection rate was also higher in DGF recipients at 6 months (6.0% vs. 4.7% p < 0.001). This difference remained through the 1-year mark (8.9% vs. 7.3% p < 0.001). BK infection rate within the first year was not different between groups (8.8% vs. 9.4%, p = 0.14) in recipients with and without DGF, respectively, although BK data was only available on 28,147 recipients (27.1%). The 1-year creatinine level was higher in the DGF group, at 1.58 mg/dL (±0.7) compared to 1.37 mg/dL (±0.6) (p < 0.001).

**TABLE 2:** Short-term outcomes by DGF status.

Outcome	No DGF (N = 72,481)	DGF (N = 31,197)	p value
Six-month rejection^1^	3,140 (4.7%)	1,615 (6.0%)	<0.001
One-year rejection	4,601 (7.3%)	2,180 (8.9%)	<0.001
Length of stay (Days)	5.7 (10.15)	8.2 (12.0)	<0.001
One-year re-hospitalization	28,812 (45%)	1,308 (54.1%)	<0.001
BK infection^2^	1970 (9.4%)	639 (8.8%)	0.141
One-year creatinine (mg/dL)	1.37 (0.59)	1.58 (0.71)	<0.001

Values are presented as mean (SD) for continuous variables and as n (%) for categorical variables. P-values were calculated using t-tests for continuous variables and chi-square tests for categorical variables.

Abbreviations: DGF, delayed graft function; BK, BK, polyomavirus.

1 Rejection is defined as biopsy-proven or treated rejection.

2 BK, virus data available through 2014 only.

**FIGURE 3:**
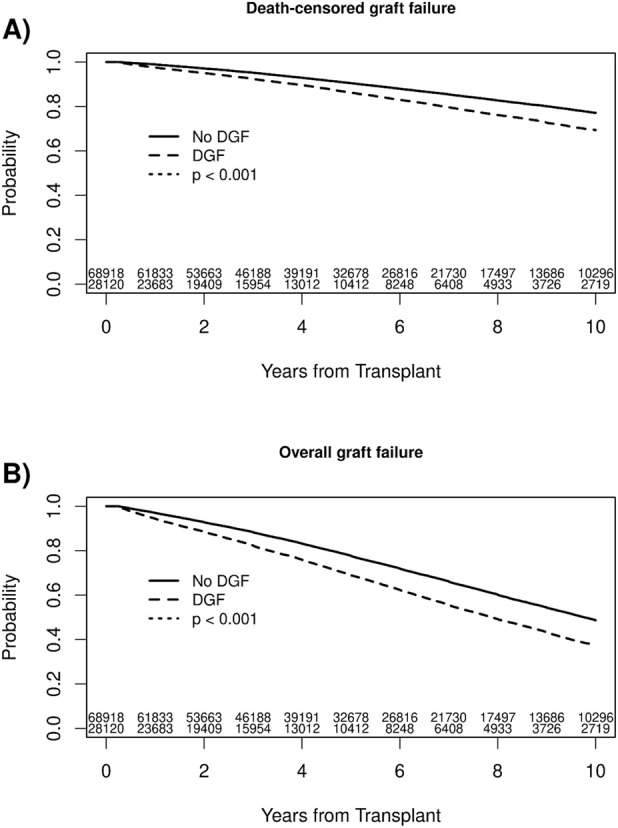
**(A)** Death-censored graft survival and **(B)** overall graft survival by delayed graft function (DGF) status. Kaplan–Meier curves show significantly lower graft survival among recipients with DGF than among those without DGF (log-rank p < 0.001 for both outcomes). At 5 and 10 years, death-censored graft survival was 77.7% and 48.7% in recipients without DGF versus 68.9% and 37.4% in those with DGF, respectively. Corresponding overall graft survival at 5 and 10 years was 72.6% and 41.2% in recipients without DGF versus 63.1% and 31.8% in those with DGF, respectively.

In the Kaplan-Meier analysis, DGF was associated with worse overall and death-censored graft survival (both log-rank p < 0.001) (Figure 3). Overall graft survival at 5 and 10 years in patients without DGF was 77.7% (CI 77.3, 78.0) and 48.7% (CI 48.1, 49.2) vs. 68.9% (CI 68.3, 69.6) and 37.4% (CI 36.5, 38.3) in those with DGF, respectively.

### Adjusted outcomes

To further examine the impact of DGF, an adjusted analysis was performed. In the Cox proportional hazards model, DGF was associated with a 40% increased risk of death-censored graft failure [HR 1.40, CI (1.33,1.47), p < 0.001] and a 27% increased risk of overall graft loss by 10 years post-transplant [HR 1.27, CI (1.23,1.31), p < 0.001]. Older recipient age was associated with lower death-censored graft failure [HR 0.97, CI (0.97, 0.97), p < 0.001] but higher overall graft failure [HR 1.02, CI (1.02, 1.02), p < 0.001]. Death-censored and overall graft failure were slightly lower in recipients of Hispanic or other ethnicities compared to white recipients. Black recipients had an increased risk of death-censored graft failure [HR 1.42, CI (1.34, 1.51), p < 0.001], but not overall graft failure [HR 0.99, CI (0.95, 1.02), p = 0.42] compared to white recipients. Compared to diabetes as a cause of ESRD, other ESRD etiologies were associated with lower death-censored and overall graft failure (Table 3).

**TABLE 3:** Cox proportional hazard models for death-censored and overall kidney graft failure by DGF status.

Variable	Death-censored graft failure	Overall graft failure
​	[HR, (95% C.I), p-value]	[HR, (95% C.I), p-value]
DGF	[HR 1.40 (1.33, 1.47), p < 0.01]	[HR 1.27, (1.23, 1.31), p < 0.01]
Recipient age	[HR 0.97 (0.97, 0.97), p < 0.01]	[HR 1.02, (1.02, 1.02), p < 0.01]
Recipient sex (male)	[HR 1.05, (0.99, 1.10), p = 0.08]	[HR 1.09, (1.06, 1.13), p < 0.01]
Ethnicity		
White	Ref	Ref
Black	[HR 1.42 (1.34, 1.51), p < 0.01]	[HR 0.99, (0.95, 1.02) p = 0.42]
Hispanic	[HR0.86, (0.80, 0.93), p < 0.01]	[HR 0.77, (0.74, 0.81), p < 0.01]
Other	[HR 0.87, (0.79, 0.96), p < 0.01]	[HR 0.73, (0.69, 0.78), p < 0.01]
ESKD etiology		
DM	Ref	Ref
PCKD	[HR 0.61, (0.54, 0.68), p < 0.01]	[HR 0.49, (0.46, 0.52), p < 0.01]
GN	[HR 0.87, (0.79, 0.94), p < 0.01]	[HR 0.68, (0.65, 0.71), p < 0.01]
Other	[HR 0.95, (0.9, 1.01), p = 0.08]	[HR 0.75, (0.72, 0.77), p < 0.01]
Dialysis vintage	[HR 1.00, (1, 1.01), p = 0.39]	[HR 1.03, (1.02, 1.03), p < 0.01]
Vascular disease	[HR1.06, (0.99, 1.15), p = 0.09]	[HR 1.26, (1.21, 1.31), p < 0.01]
Single kidney	Ref	Ref
*En-bloc*	[HR 0.54, (0.43, 0.68), p < 0.01]	[HR 0.63, (0.55, 0.73), p < 0.01]
Dual kidney	[HR 0.82, (0.67, 1.01), p = 0.08]	[HR 0.82, (0.73, 0.93), p < 0.01]
Local organ	[HR 0.94, (0.88, 1.01), p = 0.09]	[HR 0.99, (0.95, 1.04), p = 0.79]
KDRI	[HR 1.89, (1.68, 2.13), p < 0.01]	[HR 1.48, (1.38, 1.60), p < 0.01]
DBD	[HR 1.13, (1.05, 1.21), p < 0.01]	[HR 1.11, (1.06, 1.16), p < 0.01]
Donor age	[HR 1.01, (1.01, 1.01), p < 0.01]	[HR 1.01, (1.0, 1.01), p < 0.01]
Donor ethnicity		
White	Ref	Ref
Black	[HR 1.12, (1.04, 1.20), p < 0.01]	[HR 1.05, (1.00, 1.10), p = 0.03]
Hispanic	[HR 0.94, (0.88, 1.02), p = 0.12]	[HR 0.95, (0.91, 1.00), p = 0.04]
Other	[HR 1.15, (1.02, 1.30), p = 0.02]	[HR 1.09, (1.01, 1.18), p = 0.02]
Donor sex (male)	[HR 0.98, (0.94, 1.03), p = 0.49]	[HR 0.98, (0.95, 1.01), p = 0.12]
Pumped	[HR 1.01, (0.95, 1.07) p = 0.80]	[HR 1.00, (0.96, 1.04), p = 0.97]
Cold ischemia time	[HR 1.00, (1, 1.01) p = 0.13]	[HR 1.00, (1, 1.01), p = <0.01]
Pre-KAS	Ref	​
KAS	[HR 0.85, (0.80, 0.90), p < 0.01]	[HR 1.04, (1.00, 1.08), p = 0.04]
KAS-250	[HR 0.95, (0.72, 1.25), p = 0.72]	[HR 1.04, (0.89, 1.21), p = 0.64]
HLA-mismatch	​	​
0	Ref	Ref
1–3	[HR 1.25, (1.10, 1.41) p < 0.01]	[HR 1.09, (1.02, 1.17), p < 0.01]
4–6	[HR 1.37, (1.22, 1.53) p < 0.01]	[HR 1.13, (1.06, 1.21), p < 0.01]
cPRA	[HR 1.09, (1.01, 1.19) p = 0.04]	[HR 1.11, (1.05, 1.17), P < 0.01]
Induction type	​	​
r-ATG	Ref	Ref
IL-2RA	[HR 1.05, (0.98, 1.13) p = 0.15]	[HR 1.05, (1.01, 1.10) p = 0.02]
Alemtuzumab	[HR 1.05, (0.97, 1.14) p = 0.23]	[HR 0.99, (0.94, 1.05) p = 0.79]
Steroid maintenance	[HR 1.03, (0.96, 1.1) p = 0.47]	[HR 1.07, (1.03, 1.12) p < 0.01]

Hazard ratios (HR) with 95% confidence intervals (CI) are reported. Models were adjusted for recipient demographics, donor characteristics, immunologic factors, and procurement variables as specified in the Methods section.

Continuous variables are modeled per-unit increases: recipient age and dialysis vintage per year, KDRI, per 0.1-unit increase, and cold ischemia time per hour.

Abbreviations: HR, hazard ratio; CI, confidence interval; DGF, delayed graft function; ESKD, end-stage kidney disease; KDRI, kidney donor risk index; DBD, donation after brain death; KAS, kidney allocation system; cPRA, calculated panel reactive antibody.

Compared to the pre-KAS era, the KAS era was associated with 15% decreased death-censored graft failure [HR 0.85, CI (0.80, 0.90), p < 0.001] and 4% increased risk of overall graft failure [HR 1.04, CI (1.00,1.08), p = 0.04]. The post-KAS 250 was not associated with a higher risk of death-censored graft failure [HR 0.95, CI (0.72, 1.25), p = 0.72] or overall graft failure [HR 1.04, CI (0.89–1.21), p = 0.64].

KDRI, DBD and donor age were significantly associated with a higher risk of death censored and overall graft failure, whereas vascular disease and dialysis time were only significantly associated with the latter. Compared to rabbit Anti-thymocyte globulin (r-ATG), neither alemtuzumab nor Interleukin 2 Receptor Antagonists (IL-2RA) was associated with increased death-censored graft failure. However, IL-2RA was associated with a 5% increased risk of overall graft failure [HR 1.05, CI (1.01, 1.10), p = 0.021]. Steroid use was associated with an increased risk of overall graft failure but not death-censored kidney graft failure. Compared to conventional single kidney transplants, en-bloc transplants were associated with lower death-censored and overall graft failure; dual kidney transplants were associated with lower overall graft failure but not death-censored graft failure (Table 3).

To further explore the relationship between delayed graft function (DGF) and acute rejection, we performed stratified analyses based on rejection status within the first-year post-transplant. DGF remained significantly associated with both death-censored and overall graft failure in recipients with and without rejection. No significant interaction between DGF and rejection status was observed, suggesting that DGF’s adverse effect on long-term outcomes is independent of rejection status (Table 4).

**TABLE 4:** Cox proportional hazard models for delayed graft function and graft failure stratified by acute rejection status.

Model	Death-censored graft failure [HR, (95% C.I), p-value]	Overall graft failure [HR, (95% C.I), p-value]
Model 1 (recipients with rejection)	​	​
DGF	[HR 1.31, (1.15, 1.50); <0.01]	[HR 1.24, (1.12, 1.37); <0.01]
Model 2 (recipients without rejection)	​	​
DGF	[HR 1.28, (1.21, 1.37); <0.01]	[HR 1.21, (1.17, 1.26); <0.01]
Model 3 (all recipients)	​	​
DGF	[HR 1.3, (1.23, 1.39); <0.01]	[HR 1.22 (1.18, 1.27); <0.01]
Acute rejection	[HR 2.50, (2.30, 2.71); <0.01]	[HR 1.78, (1.67, 1.89); <0.01]
Interaction between DGF and rejection	[HR 0.92, (0.81, 1.06); 0.27]	[HR 0.98, (0.89, 1.08); 0.71]

Hazard ratios (HR) with 95% confidence intervals (CI) are reported. Models were adjusted for recipient demographics, donor characteristics, immunologic factors, and transplant-related variables consistent with the primary analysis.

Model 1: recipients with acute rejection in year 1; Model 2: recipients without rejection; Model 3: full cohort, with both delayed graft function and acute rejection as covariates, plus their interaction term.

All models were adjusted for the covariates as in the primary analysis.

Abbreviations: HR, hazard ratio; CI, confidence interval; DGF, delayed graft function.

### Outcomes among mate-kidneys cohort

**FIGURE 4:**
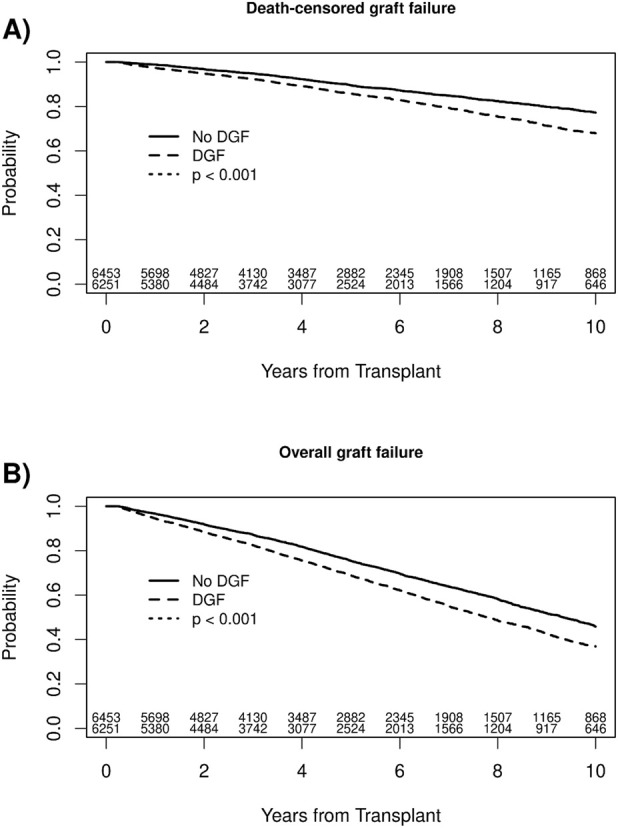
**(A)** Death-censored graft survival and **(B)** overall graft survival by delayed graft function (DGF) status among recipients of mate kidneys (same donor). Kaplan–Meier curves show significantly lower graft survival among recipients with DGF than their paired counterparts without DGF (log-rank p < 0.001 for both outcomes), confirming the independent effect of DGF after controlling for donor characteristics. At 5 and 10 years, both death-censored and overall graft survival show a persistent separation between groups, with lower survival in recipients experiencing DGF.

To control for unaccounted donor factors, we performed a mate-kidney analysis, and we found, in the unadjusted analysis, that those who experienced DGF, had lower death-censored kidney survival (log-rank p < 0.001) and overall kidney graft survival (log-rank p < 0.001), compared to those without DGF (Figure 4). In the mate-kidneys cohort with fully adjusted models (Table 5), delayed graft function (DGF) was associated with an 58% higher risk of death-censored graft failure (HR 1.58; 95% CI 1.39–1.80; p < 0.01) and a 35% higher risk of overall graft failure (HR 1.35; 95% CI 1.25–1.46; p < 0.01).

**TABLE 5:** Cox proportional hazard models for death-censored and overall kidney graft failure by DGF Status among recipients of kidney pairs from the same donor (mate kidneys).

Variable	Death-censored graft failure	Overall graft failure
​	[HR, (95% C.I), p-value]	[HR, (95% C.I), p-value]
DGF	[HR 1.58, (1.39, 1.80), p < 0.01]	[HR 1.35, (1.25, 1.46), p < 0.01]

Hazard ratios (HR) with 95% confidence intervals (CI) are reported. Models were adjusted for the same covariates as the primary analysis, including recipient demographics, immunologic factors, transplant-related variables, and additional adjustment for donor pairing.

Abbreviations: HR, hazard ratio; CI, confidence interval; DGF, delayed graft function.

## Discussion

In this national analysis of more than 100,000 deceased donor kidney recipients, we demonstrate that DGF is associated with substantially increased risks of long-term death-censored and overall graft failure, with adverse effects persisting for up to a decade after transplantation. Notably, even after restricting the analysis to mate kidneys (thereby fully controlling for donor quality) DGF remained a powerful independent predictor of inferior allograft survival. These findings highlight the lasting clinical significance of DGF well beyond the perioperative period.

Our results extend and significantly strengthen prior work. Existing literature has documented the short-term consequences of DGF, including higher rejection risk, prolonged recovery, and increased hospital utilization [11–19]. However, long-term outcomes have been inconsistently reported, limited by small sample sizes or short follow-up. The present study confirms that the detrimental effects of DGF persist well into late post-transplant years and are not explained solely by baseline donor characteristics or center-level practice variation.

Mechanistically, these findings are biologically plausible. DGF reflects significant ischemia-reperfusion injury, which induces tubular epithelial cell damage, inflammatory activation, and heightened immunogenicity [13, 20, 21]. These early injuries may predispose the graft to chronic interstitial fibrosis, subclinical allo-immunity, and progressive decline in graft function. The higher rates of early rejection, worse early creatinine, and greater rehospitalization among DGF recipients in our cohort support this mechanistic pathway. However, in our additional analyses, the association between DGF and long-term graft failure persisted irrespective of rejection status, and no significant interaction was observed. These findings suggest that while rejection contributes to worse outcomes, DGF is independently associated with sustained effect on graft survival.

The mate-kidney analysis (using kidneys from the same donor) confirmed the independent association between DGF and adverse transplant outcomes. Recipients that experienced DGF had an 80% and a 45% increased risk of long-term death-censored and overall graft failure, respectively. Again, these findings emphasize the relevance of DGF beyond the immediate post-transplant period. Moreover, the disparity observed between mate kidneys demonstrates the lasting impact of experiencing DGF on allograft survival, while also highlighting the pivotal contribution of recipient-specific factors in determining long-term transplant outcomes, independent of donor quality.

Machine perfusion (pump preservation) has been studied as a strategy to reduce the ischemia-reperfusion injury and the incidence of DGF. Some reports have demonstrated that its use is associated with a reduction in DGF [22–25], however its benefits might not be universally observed, as other studies have reported that machine perfusion was not associated with a clear benefit [26]. In our cohort, after adjusting for donor, recipient, immunologic and procurement factors, machine perfusion itself was not independently associated with improved long-term graft survival. These findings suggest that while the use of machine perfusion might provide some advantages in the short term, the long-term effect is not observed.

Our findings extend on the work by Lim et al [6] where they analyzed 74 pairs of DCD kidneys, with a median follow up of 1.9 years, and reported a greater proportion of recipients with DGF had experienced overall graft loss and death censored graft loss at 3 years compared to those without DGF. Compared to their study, our analysis leveraged a markedly larger and more diverse cohort (over 100,000 recipients including both DBD and DCD donors) thereby enhancing statistical power and generalizability. Our longer follow-up also allowed us to find late graft failures and show the sustained effect of DGF. Furthermore, by restricting our cohort to recipients with uniform maintenance immunosuppression and applying a mixed-effects Cox model with center-level random effects we were able to minimize confounding from treatment heterogenicity and practice variation.

The Kidney Allocation System (KAS) was designed to improve allocation equity [6]; however, our findings highlight the increasing prevalence of DGF over our study period, particularly after the implementation of the KAS in 2014 and KAS250 in 2021, consistent with previous reports [27–29]. The reasons behind this trend are likely multifactorial and probably reflect continuous changes in donor and recipient selection criteria, the increased utilization of donation after circulatory death (DCD) organs (which are more prone to ischemia-reperfusion injury), as well as other logistical factors prolonging ischemia time [29, 30].

### Strengths and limitations

This study has several limitations inherent to its retrospective, registry-based design. Despite adjustment for a comprehensive set of donor, recipient, immunologic, and procurement variables, residual confounding from unmeasured or incompletely captured factors may persist, and causal inferences cannot be definitively established. Although the mate-kidney analysis strengthens causal inference by controlling for donor-related factors, it does not account for center-level practices or recipient-specific clinical decisions that may influence outcomes.

Delayed graft function (DGF) was defined using the standard SRTR/OPTN definition (dialysis within the first week post-transplant); however, this definition does not capture the severity or duration of DGF, as the registry records it as a dichotomous variable. As such, we were unable to evaluate potential dose-response relationships between DGF severity and long-term outcomes. In addition, variability in clinical practice, particularly differences in dialysis initiation thresholds across centers, may lead to misclassification of DGF and introduce bias.

The SRTR also lacks granular data on immunosuppression management, including the timing of calcineurin inhibitor initiation, dosing, and drug levels. Therefore, we could not assess whether delayed or subtherapeutic tacrolimus exposure contributed to the higher rejection rates or worse outcomes observed in recipients with DGF.

Despite these limitations, our study has several and notable strengths. It includes a large cohort of transplant recipients, long-term follow-up and comprehensive reporting of outcomes and risk factors. Most importantly, the subgroup analysis of mate kidneys allowed us to control for donor-related confounders. Finally, the large sample size provided enough statistical power to detect statistically significant differences.

### Conclusion

DGF is a strong, independent, and durable predictor of inferior long-term kidney allograft outcomes. Importantly, the association persists even after controlling for all donor-related factors using mate-kidney analysis. As DGF continues to rise in the current allocation era, strategies focused on prevention, early identification, and enhanced post-transplant management are critical to improving long-term outcomes for deceased donor kidney recipients.

## Data Availability

The original contributions presented in the study are included in the article/supplementary material, further inquiries can be directed to the corresponding author.
